# Interpretando o Infarto Agudo do Miocárdio 40 Anos Depois. Evolução do Conhecimento: Qual a Melhor Explicação?

**DOI:** 10.36660/abc.20230757

**Published:** 2024-04-15

**Authors:** Vitor Coutinho Andrade, Paulo Cury Rezende, Whady Hueb, José Antonio Franchini Ramires

**Affiliations:** 1 Universidade de São Paulo Faculdade de Medicina Instituto do Coração do Hospital das Clínicas São Paulo SP Brasil Instituto do Coração do Hospital das Clínicas da Faculdade de Medicina da Universidade de São Paulo, São Paulo, SP – Brasil

**Keywords:** Infarto do Miocárdio, Miocárdio Atordoado, Vaso Espamo Coronário, Dislipidemias, Diagnóstico por Imagem/métodos

## Introdução

Ruptura e erosão da placa são as patologias imediatas mais comuns associadas ao infarto agudo do miocárdio. No entanto, outros mecanismos devem ser considerados. O caso relatado ocorreu em um momento em que a revascularização coronária percutânea ou farmacológica não era o tratamento padrão, levando à discussão sobre esses outros mecanismos, bem como ao possível benefício atribuído ao procedimento de revascularização miocárdica.

## Descrição

**Figura 1:**
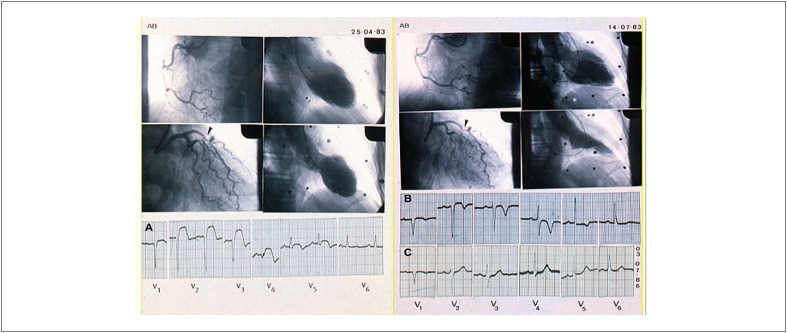
Imagens de cineangiocoronariografia e ECG imediata e tardia. Quadro superior esquerdo: Suboclusão da artéria descendente anterior. Artérias circunflexa e coronária direita: ausência de obstruções. Ventrículo esquerdo: disfunção sistólica grave. Quadro superior direito (três meses depois). Angiografia semelhante com recuperação do ventrículo esquerdo. ECG (A): supradesnivelamento do segmento ST nas derivações precordiais. ECG (B): apenas recuperação do segmento ST e inversão da onda T. ECG (C): Três anos depois: próximo ao normal.

Homem de 58 anos, previamente assintomático e portador de hipertensão arterial e dislipidemia, foi avaliado no pronto-socorro (25/março/1983) com dor retroesternal típica com irradiação para o braço esquerdo, acompanhada de sudorese e palidez, iniciados 1 hora atrás. Na apresentação, os sinais vitais eram estáveis e o exame físico era normal. A hipótese principal foi de infarto agudo do miocárdio (IAM), corroborada pelo eletrocardiograma (ECG) de 12 derivações que mostrava supradesnivelamento do segmento ST com posterior formação de onda Q nas derivações precordiais ( Figura 1 ). A coronariografia mostrou um padrão uniarterial com suboclusão da artéria descendente anterior e disfunção ventricular esquerda grave ( Figura 1 ). Os exames laboratoriais são apresentados na Tabela 1 . A dor torácica foi aliviada com o uso de nitroglicerina endovenosa e analgesia com análogo de morfina. Após o período de recuperação, o paciente recebeu alta hospitalar.

**Tabela 1:** Características laboratoriais na admissão

**Hemoglobina**		14 g/dL
**Hematócrito**		43%
**Leucócitos**		6,510 mm³
**Sódio**		142 mEq/L
**Potássio**		4,1 mEq/L
**Ureia**		41 mg/dL
**Creatinina**		0,9mg/dL
**PCR**		<5 mg/L
**CK-MB**		988 U/L
**Gasometria Arterial**		
	pH	6,98
	pCO_2_	16,02 mmHg
	pO_2_	21,70 mmHg
	HCO_3_	27,8 mEq/L
	Excesso de base	-7,5 mEq/L
	Lactato	2,7 mg/dL

Três meses depois, (14/julho/1983), a paciente retornou à emergência com sintomas anginosos, mas sem sudorese ou palidez. Na apresentação, os sinais vitais também estavam estáveis e o exame físico inalterado. O ECG mostrava recuperação do segmento ST e apenas inversão da onda T, além das mesmas características angiográficas encontradas no exame inicial (persistência do padrão uniarterial com suboclusão proximal da artéria descendente anterior), exceto pela ventriculografia demonstrando recuperação da função ventricular ( Figura 1 ). Recebeu alta hospitalar com seguimento ambulatorial, onde persistiu sem sintomas anginosos ou sinais de insuficiência cardíaca por anos. Em 1986, três anos após o evento índice, realizou novo ECG em caráter ambulatorial, que demonstrou normalização do mesmo, com ausência de onda Q em derivações anteriores, sugerindo ausência de fibrose ventricular nesta região.

## Discussão

Sabe-se que a ruptura e a erosão da placa são as patologias imediatas mais comuns associadas ao IAM. No entanto, outros mecanismos devem ser considerados:

### A- Espasmo da artéria coronária

O IAM por espasmo é subdiagnosticado e refere-se a uma vasoconstrição súbita e intensa de uma artéria coronária epicárdica que causa oclusão ou quase oclusão do vaso que, se prolongada, pode evoluir para lesão miocárdica irreversível. Os mecanismos propostos incluem disfunção endotelial e hiper-reatividade primária das células musculares lisas vasculares. No presente caso, a possibilidade de um mecanismo de espasmo coronariano não deve ser ignorada.

### B- IAM com artéria "fechada/aberta"

Muitas evidências confirmaram a fissura da placa aterosclerótica seguida pela formação de trombo como o processo patológico subjacente que causa a oclusão da artéria coronária, resultando em IAM. DeWood et al.,^1^ demonstraram a ocorrência de trombo oclusivo em 86% dos pacientes na angiografia dentro de 4 horas após o início do IAM e essa proporção diminuiu significativamente para 65% quando os pacientes foram estudados 12 a 24 horas após o início dos sintomas. A oclusão coronariana resulta em isquemia miocárdica, levando a disfunção ventricular e necrose miocárdica. A restauração espontânea do fluxo anterógrado durante este período inicial do IAM pode interromper a progressão da morte celular miocárdica e salvar a função no miocárdio comprometido. Nesse caso, a lise espontânea e precoce do trombo parece ser uma possibilidade.

### C- Trombólise e reperfusão espontâneas.

Em procedimento pioneiro, Galiano et al.,^2^ realizaram reperfusão mecânica na fase aguda do IAM. Após identificar um trombo na artéria coronária direita de um paciente com choque cardiogênico, o cardiologista intervencionista "fraturou" o trombo oclusivo, proporcionando a reperfusão e a reversão do choque cardiogênico. Durante a angiografia realizada na fase aguda, não foi observado nenhum trombo intraluminal. No entanto, a ocorrência de trombólise espontânea e reperfusão parece ser um modelo racional de oclusão coronariana completa rapidamente aliviada por mecanismos trombolíticos intrínsecos, que podem ser difíceis de diagnosticar pela angiografia, mas não podem ser descartados.

### D-Síndrome de Takotsubo

A cardiomiopatia de estresse ocorre em aproximadamente 1 a 2 por cento dos pacientes que apresentam IAM com supradesnivelamento do segmento ST.^3^ A patogênese desse distúrbio não é bem compreendida. Várias características da cardiomiopatia de estresse sugerem que esse distúrbio pode ser causado por espasmo ou disfunção microvascular difusa induzida por catecolaminas, resultando em atordoamento miocárdico.^4^ ou por toxicidade miocárdica direta associada a catecolaminas.^5^ O suporte para um possível papel patogênico das catecolaminas vem de estudos nos quais as catecolaminas plasmáticas foram medidas na apresentação.^6^ No presente caso, a possibilidade de cardiomiopatia de Takotsubo também não foi descartada, embora menos provável após a evidência angiográfica de doença arterial coronariana obstrutiva.

### E- Miocárdio atordoado

O atordoamento do miocárdio foi estabelecido nos experimentos pioneiros de Heyndrickx et al.,^7^ que demonstraram em modelo canino que uma breve oclusão coronária era seguida de disfunção contrátil pós-isquêmica regional sustentada que se recuperava totalmente após horas, sendo essa recuperação mais lenta naqueles expostos a maior tempo de isquemia.

Assim, o entendimento do miocárdio atordoado caracteriza-se por uma disfunção contrátil desproporcionalmente duradoura (horas a dias), porém reversível, que se segue a um breve surto de isquemia miocárdica quando o fluxo sanguíneo coronariano é totalmente restaurado, compatível com a recuperação ventricular evidenciada neste caso.

## Mensagem Final

O caso relatado ocorreu em um momento em que a revascularização coronária percutânea não era o tratamento padrão na síndrome coronariana aguda. Apenas em 1986, com a publicação do estudo GISSI,^8^ inaugurou-se a era da reperfusão química. Assim, a recuperação da função ventricular ocorreu mesmo com a limitação do fluxo coronariano causada pela suboclusão da artéria. Atualmente, a recuperação ventricular observada neste caso provavelmente seria atribuída à intervenção de restauração do fluxo coronariano, porém este raciocínio não é sempre correto. Além disso, ao observar uma placa coronária suboclusiva, devemos sempre questionar a ocorrência de mecanismos não ateroscleróticos adjuvantes para a ocorrência de síndrome coronariana aguda. Como mostrado acima, apesar de vários mecanismos fisiopatológicos possíveis, defini-la nem sempre é uma tarefa fácil.
