# Supplementary material for: Interpretando o Infarto Agudo do Miocárdio 40 Anos Depois. Evolução do Conhecimento: Qual a Melhor Explicação?
Source: Arq Bras Cardiol. 2024 Apr 15;121(3):e20230757. [Article in Portuguese] doi: 10.36660/abc.20230757 (PMC11081065; doi:10.36660/abc.20230757)
Supplement: Translation

# Translation: Interpreting Acute Myocardial Infarction 40 Years Later. Evolution of Knowledge: What is the Best Explanation?

**DOI:** 10.36660/abc.2023-0757i

**Authors:** Vitor Coutinho Andrade, Paulo Cury Rezende, Whady Hueb, José Antonio Franchini Ramires

**Affiliations:** 1 São Paulo SP Brazil Instituto do Coração do Hospital das Clínicas da Faculdade de Medicina da Universidade de São Paulo, São Paulo, SP – Brazil

**Keywords:** Myocardial Infarction, Myocardial Stunning, Coronary Vasoespasm, Dyslipidemias, Diagnostic, Imaging/methods

## Introduction

Plaque rupture and erosion are the most common immediate pathologies associated with acute myocardial infarction. However, other mechanisms must be considered. The reported case occurred at a time when percutaneous or pharmacological coronary revascularization were not the standard treatment, which sparked discussion about these other mechanisms, as well as the possible advantages associated with the myocardial revascularization procedures.

## Description

A 58-year-old man, previously asymptomatic and diagnosed with arterial hypertension and dyslipidemia, was evaluated in the emergency room (March 25, 1983) with severe retrosternal chest pain radiating to the left arm, accompanied by sweating and pallor, which had begun one hour before he arrived at the hospital. On presentation, vital signs were stable, and physical examination was unremarkable. The primary diagnosis was acute myocardial infarction (MI), supported by a 12-lead electrocardiogram (ECG) showing ST-segment elevation with subsequent formation of Q waves in precordial leads ( Figure 1 ). Coronary angiography revealed a single-vessel pattern with subtotal occlusion of the left anterior descending artery and severe left ventricular dysfunction ( Figure 1 ). Laboratory findings are presented in Table 1 . Chest pain was relieved with intravenous nitroglycerin and analgesia with a morphine analog. Following a recovery period, the patient was discharged.

**Figure 1:** Immediate and late coronary cineangiography and ECG images. CAPTION: Upper left frame: Sub occlusion of the anterior descending artery. Circumflex and right coronary arteries: absence of obstructions. Left ventricle: severe systolic dysfunction. Upper right frame (three months later). Similar angiogram with recovery of the left ventricle function. ECG (A): ST-segment elevation in precordial leads. ECG (B): ST-segment recovery and T wave inversion only. ECG (C): Three years later: Close to normal.

**Table 1:** Laboratory characteristics on admission

**Hemoglobin**		14 g/dL
**Hematocrit**		43%
**White cells count**		6.510 mm^3^
**Sodium**		142 mEq/L
**Potassium**		4.1 mEq/L
**Urea**		41 mg/dL
**Creatinine**		0.9mg/dL
**C reactive protein**		<5 mg/L
**CK-MB (peak)**		988 U/L
**Arterial blood gas**		
	pH	6,98
	pCO_2_	16.02 mmHg
	pO_2_	21.70 mmHg
	HCO_3_	27.8 mEq/L
	Base excess	-7.5 mEq/L
	Lactate	2.7 mg/dL

Three months later (July 14, 1983), the patient returned to the emergency department with anginal symptoms, but without sweating or pallor. On presentation, vital signs were again stable, and the physical examination was unchanged. The ECG showed recovery of the ST segment and only T wave inversion, along with the same angiographic characteristics as the initial examination (persistent single-vessel pattern with proximal sub occlusion of the left anterior descending artery), except for ventriculography demonstrating recovery of ventricular function ( Figure 1 ). The patient was discharged with outpatient follow-up, remaining asymptomatic for angina or signs of heart failure for years. In 1986, three years after the index event, a subsequent ambulatory ECG showed normalization, with the absence of Q waves in anterior leads, suggesting the absence of ventricular fibrosis in this region.

## Discussion

It is known that plaque rupture and erosion are the most common immediate pathologies associated with acute MI. However, other mechanisms must be considered:

### A- Coronary artery spasm

MI due to spasm is underdiagnosed and refers to a sudden and intense vasoconstriction of an epicardial coronary artery that causes vessel occlusion or near occlusion that, if prolonged, can progress to irreversible myocardial injury. Proposed mechanisms include endothelial dysfunction and primary hyperreactivity of vascular smooth muscle cells. In the present case, the possibility of a coronary spasm mechanism should not be ignored.

### B- Acute MI with "close/open" artery

Much evidence has confirmed atherosclerotic plaque fissuring followed by thrombus formation as the underlying pathologic process causing coronary artery occlusion resulting in acute MI. DeWood et al.^1^showed occlusive thrombus in 86 percent of the patients in angiography within 4 hours of the onset of acute MI and this proportion decreased significantly to 65 percent when patients were studied 12 to 24 hours after the onset of symptoms. Coronary occlusion results in myocardial ischemia, leading to ventricular dysfunction and myocardial necrosis. Spontaneous restoration of antegrade flow during this early period of acute MI can arrest the progression of myocardial cell death and salvage function in jeopardized myocardium. In this case, spontaneous and early lysis of the thrombus seems to be an attractive possibility.

### C- Spontaneous thrombolysis and reperfusion

In a pioneering procedure, Galiano et al.^2^performed mechanical reperfusion in the acute phase of MI. After identifying a thrombus in the right coronary artery of a patient with cardiogenic shock, the interventional cardiologist "fractured" the occlusive thrombus, providing reperfusion and reversing the cardiogenic shock. During the angiography performed in the acute phase of the myocardium, no intraluminal thrombus was observed. However, the occurrence of spontaneous thrombolysis and reperfusion seems to be a rational model of complete coronary occlusion quickly relieved by intrinsic thrombolytic mechanisms, which can be difficult to diagnose by angiography but cannot be ruled out.

### D- Takotsubo Syndrome

Stress cardiomyopathy occurs in approximately 1 to 2 percent of patients presenting with ST-elevation MI.^3^The pathogenesis of this disorder is not well understood. Several features of stress cardiomyopathy suggest that this disorder may be caused by diffuse catecholamine-induced microvascular spasm or dysfunction, resulting in myocardial stunning,^4^or by direct catecholamine-associated myocardial toxicity.^5^Support for a possible pathogenic role for catecholamines comes from studies in which plasma catecholamines were measured at presentation.^6^In the present case, the possibility of Takotsubo cardiomyopathy could also not be ruled out, although less likely after the angiographic evidence of obstructive coronary artery disease.

### E- Stunning Myocardium

Myocardial stunning was established in the pioneering experiments of Heyndrickx et al.^7^, who demonstrated in a canine model that brief coronary occlusion was followed by sustained regional post-ischemic contractile dysfunction that fully recovered after hours, with this recovery being slower in those exposed to longer ischemia time.

So, the understanding of the stunned myocardium is characterized by a disproportionately long-lasting (hours to days), yet reversible, contractile dysfunction that follows a brief bout of myocardial ischemia when coronary blood flow is fully restored, compatible with the ventricular recovery evidenced in this case.

## Final message

The reported case occurred at a time when percutaneous coronary revascularization was not the standard treatment for acute coronary syndrome. It was only in 1986, with the publication of the GISSI^8^study, that the era of chemical reperfusion began. Thus, ventricular function recovery occurred despite coronary flow limitation caused by artery subocclusion. Presently, ventricular recovery observed in this case would likely be attributed to coronary flow restoration intervention; however, this reasoning is not always accurate. Additionally, when observing a subocclusive coronary plaque, we must always question the occurrence of adjunct non-atherosclerotic mechanisms for acute coronary syndrome. As demonstrated above, despite several possible pathophysiological mechanisms, defining it is not always an easy task.
